# A facile synthesis of contorted spirobisindane-diamine and its microporous polyimides for gas separation†

**DOI:** 10.1039/c7ra12719g

**Published:** 2018-02-07

**Authors:** Binod Babu Shrestha, Kazuki Wakimoto, Zhenggong Wang, Ali Pournaghshband Isfahani, Tomoya Suma, Easan Sivaniah, Behnam Ghalei

**Affiliations:** Institute for Integrated Cell-Material Sciences (iCeMS), Kyoto University 606-8501 Kyoto Japan esivanah@icems.kyoto-u.ac.jp bghalei@icems.kyoto-u.ac.jp

## Abstract

Microporous polyimides (PIM-PIs, KAUST-PIs) and polymers containing Tröger's base (TB) derivatives with improved permeability and selectivity have great importance for separation of environmental gas pairs. Despite the tremendous progress in this field, facile synthesis of microporous polymers at the industrial scale *via* designing new monomers is still lacking. In this study, a new potential approach for large scale synthesis of spirobisindane diamine (DAS) (3) has been reported from commercially available 5,5′,6,6′-tetrahydroxy-3,3,3′,3′-tetramethyl-1,1′-spirobisindane (TTSBI) and 3,4-difluoronitrobenzene. A series of DAS diamine based microporous polyimides were also synthesized. The resulting polymer membranes showed high mechanical and thermal properties with tunable gas separation performance.

## Abstract 2

Contorted diamine monomers are the attractive precursors for the synthesis of novel microporous polyimides with high gas separation performance.

## Graphical abstract

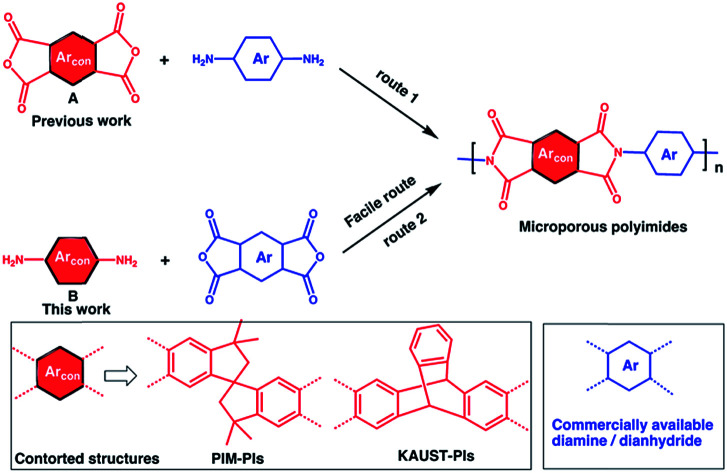

Polymeric gas separation membranes have promising applications in hydrogen recovery (H_2_/N_2_, H_2_/CH_4_), natural gas sweetening, air separation (O_2_/N_2_) and carbon dioxide purification (CO_2_/N_2_, CO_2_/CH_4_) due to their cost-effectiveness and potential environmental benefits.^1–5^ The ideal membranes should show both high permeability and selectivity. However, there is an undesirable trade-off between permeability (*P*) and selectivity (*α*) of the polymeric membrane as illustrated by the Robeson's upper bounds.^6^ According to the theoretical analysis, high permeability and selectivity can be obtained by designing shape persistent polymers having a stiff backbone.^7^ To achieve this goal, several new structures have been designed in the past.^8–11^ Among them, polymers of intrinsic microporosity (PIMs) have attained much interest in the membrane field.^12^ The main reason for this immense interest in PIMs are their rigid backbone consisting of a ladder-like repeating unit with a spirobisindane (SBI) contorted structure that provides excellent gas separation performance.^13–18^ Polyimides (PIs) are also well known materials for gas separation owing to their exceptional thermal, mechanical and chemical stability, processability, structural tunability and high pair gas selectivities. However, most conventional PIs suffer from low fractional free volume (FFV) and consequently low gas permeabilities that need to be improved in large scale practical applications.^19,20^

To address this issue, several types of microporous polyimides (PIM-PIs, KAUST-PIs) including polymers containing Tröger's base functional group with high gas separation performance, good mechanical and thermal properties have been reported (Scheme 1).^21–31^ Specifically, PIM-PIs and KAUST-PIs were generally prepared from contorted dianhydride (A) and commercially available diamine in which the main emphasis is given to the use of contorted dianhydride monomer and an aromatic diamine in PIs synthesis (Route 1). However, there are some limitations in the reported synthesis pathways, such as lengthy steps and costly reagents.^21–24^ It is more beneficial if an alternative route can avoid these difficulties. Hence, this work reveals the benefits of generating microporous polyimides from a combination of a contorted diamine (B) and an aromatic dianhydride (Route 2).

**Scheme 1:**
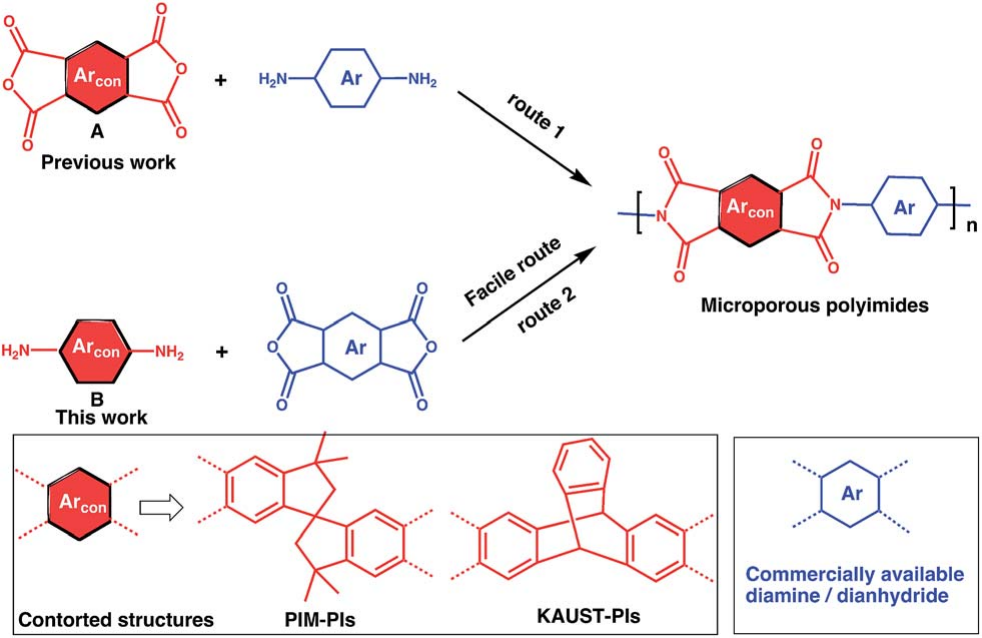
SBI-based microporous polyimides and their synthetic strategies.

Contorted diamine monomers are the attractive precursors for the synthesis of novel microporous polyimides such as TB-polyimides,^25–29^ 6FDA-DATRI^32^ and 6FDA-SBF^33^ with high gas separation performance. Also, they are versatile precursors for the synthesis of other microporous polymers such as PIM-SBI-TB.^34^ The gas separation performance can be tuned up by further functionalization of diamine. For example, *ortho* functionalized SBF diamine based PIs were found to have higher gas permeabilities than their pristine analogues due to ineffective packing caused by steric hindrance of *ortho* functional groups.^33^ In these regards, the monomers synthesized from Route 2 (contorted diamine) might have more versatility than ones from Route 1 (contorted dianhydride) for making microporous polymers. However, facile synthesis of contorted diamines has been received less attention because of the difficulty in selective amine functionalization at desired positions such as in iptycene and SBI-based diamine. Also, their reported diamine synthetic methods suffer from unwanted byproducts which need timely chromatographic purification procedure.^34–36^ Hence, designing a cost-effective facile method for novel contorted diamine monomers and thence synthesizing microporous polymer are indispensable. Herein, we propose a versatile method for SBI-diamine precursor synthesis as an efficient pathway for preparing PIM-polyimides, KAUST-polyimides and PIM-SBI-TB polymers having exceptional mechanical and thermal resilience for gas separation membrane.

In this regard, we synthesized DAS monomer (3) from commercially available TTSBI (1) and 3,4-difluoronitrobenzene. The first step includes nucleophilic substitution reaction resulting in a dinitro-SBI derivative (2). Without requiring any further purification step, it was successfully reduced to the corresponding amine (3) by palladium/carbon (Pd/C) Scheme 2 in good yields (84%). The compounds obtained in each step were characterized by ^1^H NMR, ^13^C NMR, FT-IR and MALDI Mass (ESI†). It is noteworthy that this facile method has potential application in the large-scale synthesis using 3,4 difluoronitrobenzene as a starting material; moreover, the product can be obtained in excellent yield and pure form without any additional purification step.

**Scheme 2:**
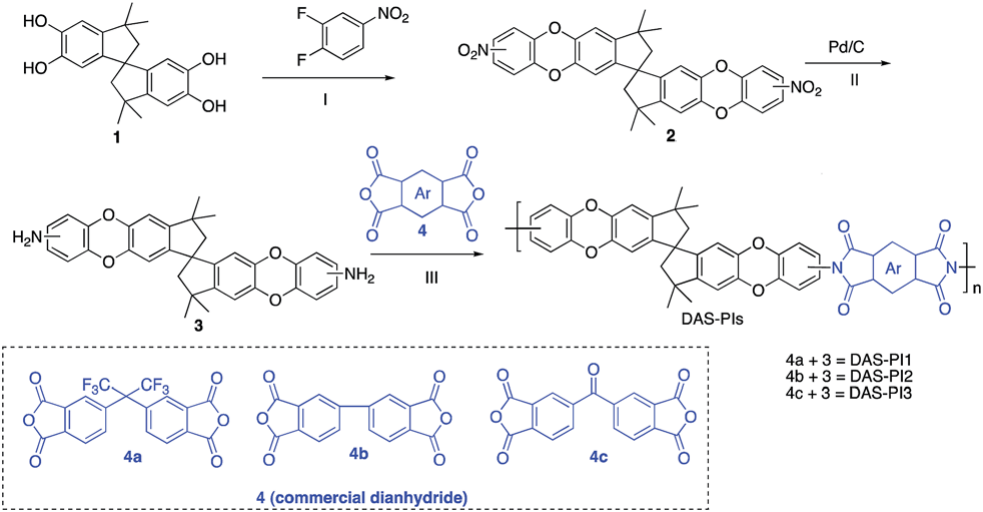
Synthesis of monomer DAS (3) and its polyimides (DAS-PIs): (I) nucleophilic substitution, (II) reduction by Pd/C, (III) one step polymerization *via* solvothermal azeotropic imidization.

In order to compare the geometric structure of SBI unit of newly synthesized diamine (DAS-diamine monomer, 3) and reported SBI-dianhydride monomer (An1).^21^ The ground state geometric structures of both monomers were fully optimized by the density hybrid function (B3LYP) method with 6-31G(d) as basis set and calculations were carried out using Gaussian 09 software package. Their geometric optimization conformations are shown in Fig. 1. The introduction of amine had almost no effect on the conformation of the SBI unit. In other words, both monomers exhibited a contortion angle of around 90° (Fig. S1†). This result indicates that DAS-diamine monomer is as equally good a precursor for synthesizing microporous polyimides as SBI-dianhydride monomers.

**Fig. 1:**
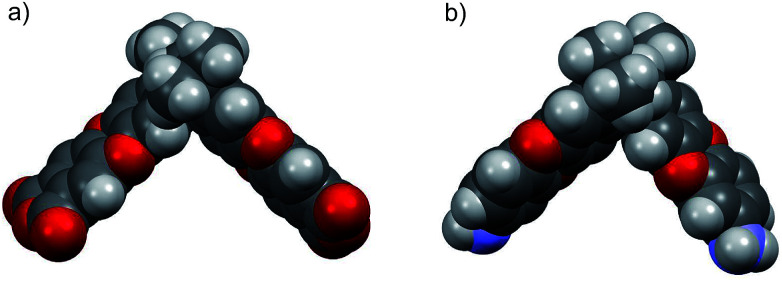
Space filling model obtained by DFT calculation (a) SBI-dianhydride (An1) (b) DAS-diamine monomer (3).

DAS diamine (3) is a versatile precursor for the synthesis of different polymers such as PIM-PIs, KAUST-PIs and PIM-SBI-TB. This time, we focused on polyimides synthesis from DAS-diamine and commercially available dianhydride. Various dianhydrides were selected (4), rigid dianhydride structure such as (hexafluoroisopropylidene)-diphthalic anhydride (6FDA) (4a) and flexible dianhydride such as biphenyl dianhydride (BPDA) (4b), benzophenone dianhydride (BTDA) (4c) are considered (Scheme 2) to investigate their effects on the polymer properties. One-step polymerization was employed *via* solvothermal azeotropic imidization reaction between DAS diamine (3) and commercially available dianhydrides (4). The obtained polyimides are called DAS-PI1, DAS-PI2 and DAS-PI3, respectively which are derived from 6FDA (4a), BPDA (4b), and BTDA (4c) (Scheme 2). The spectroscopic data and structure of monomers revealed that location of five membered imide rings and connection of C–N bond in newly synthesized PIs are different from the reported structure of PIM-polyimides (compared structures are in Fig. S1†). The synthesized polymers, DAS-PIs had high enough molecular weights, with good solubility in organic solvents to form membranes. The molecular weights and polydispersity (PDI) values of the synthesized polymers are determined by gel permeation chromatography (Table S1†). For example, DAS-PI1 has high molecular weight (*M*_w_ = 9.5 × 10^4^ g mol^−1^) and narrow PDI (1.4). In addition, their solubility charts in common organic solvents are shown in Table S2.†

To understand molecular packing and microporosity of synthesized polymers, the nitrogen adsorption and desorption behaviours were performed at 77 K. Also, the pore size distributions calculated from the N_2_ adsorption data by the Horvath–Kawazoe method are shown in Fig. 2. Rapid nitrogen uptakes were observed at very low relative pressures in the isotherms, indicating typical microporous behaviour.^37^ Changing the dianhydride parts of DAS-PI polyimides from 6FDA to BTDA resulted in significant reductions in BET surface area (Table 1). As expected, the polyimide containing 6FDA has higher free volume due to the bulky –C(CF_3_)_2_– units as compared with polyimides derived from BPDA and BTDA. To our delight, DAS-PI1 exhibited high BET surface area (345 m^2^ g^−1^), which is higher than other reported microporous poly(amine-imide)s^38^ and PIs derived from the 6FDA, such as 6FDA-DATRI^32^ (68 m^2^ g^−1^), PIM-6FDA-OH^39,40^ (225 m^2^ g^−1^) and 6FDA-SBF^33^ (240 m^2^ g^−1^). It inferred that the proper combination of rigid dianhydride and DAS-diamine leads to high surface area polyimides.

**Fig. 2:**
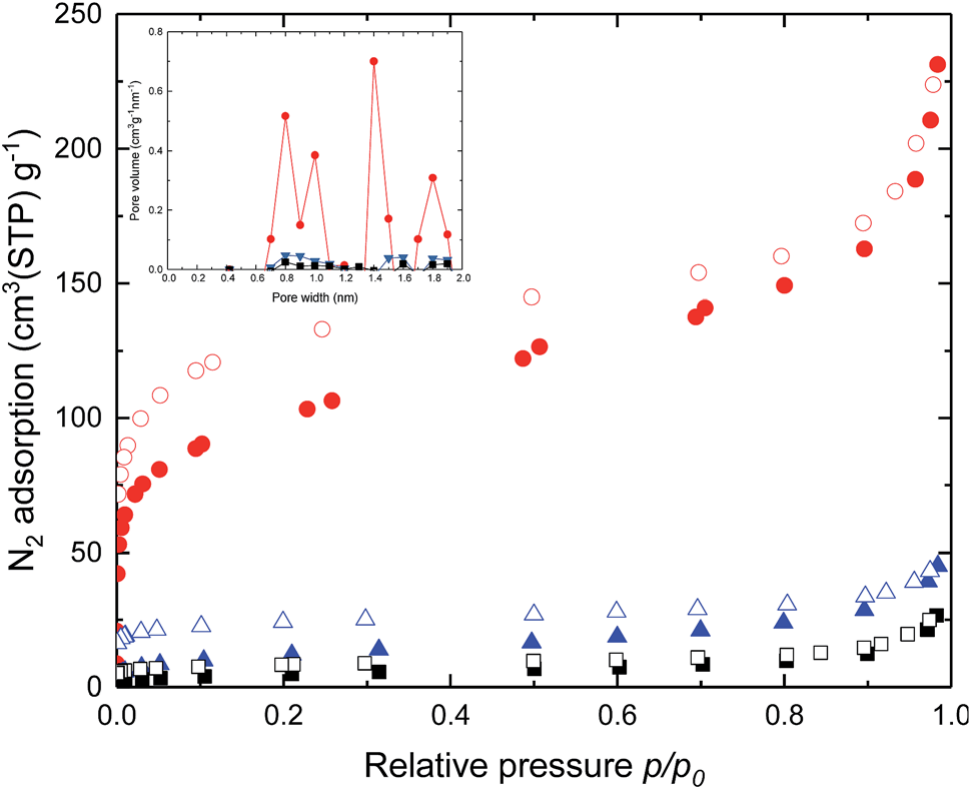
N_2_ adsorption (filled), desorption (empty) isotherms and inset is pore size distributions for the DAS-PI1 (red), DAS-PI2 (blue) and DAS-PI3 (black) at 77 K.

**Table 1:** BET surface area of DAS-PIs polymer and their corresponding monomers

Polymer	Monomers (3 + 4)	*S*_BET_ (m^2^ g^−1^)
DAS-PI1	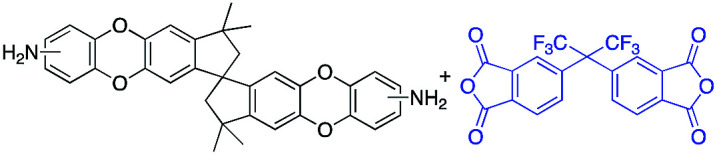	345
DAS-PI2	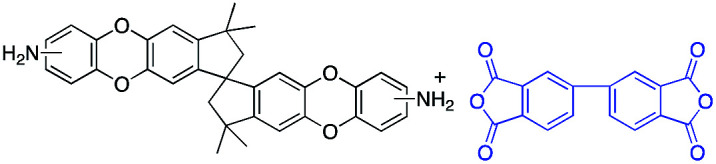	43
DAS-PI3	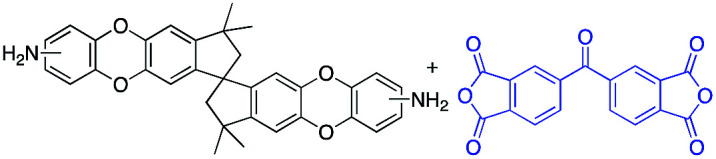	17

XRD measurements were performed to analyze the polymer inter-chain packing in DAS-PI membranes (Fig. S2†). The peak positions and corresponding *d*-spacing values are listed in Table 2. Broad peaks were observed in all XRD patterns suggesting a generally amorphous structure of DAS-PIs polyimides. The DAS-PI1 membrane showed central broad peak at 2*θ* = 15.15° along with one small broad peak located at 2*θ* = 25.04°. The calculated *d*-spacing values from given 2*θ* are 5.84 and 3.55 Å. DAS-PI2 *d*-spacing value is observed at 5.36 Å calculated from broad peak at 16.5° diffraction angle. Finally, in DAS-PI3, broad peak at 5.22 Å is calculated from 2*θ* = 16.94°. It could be inferred that DAS-PI1 has the larger *d*-spacings due to unfavorable backbone packing as compared to DAS-PI2 and DAS-PI3.

**Table 2:** Physical properties of DAS-PI polyimides^a^

Polymer	2*θ* (°)	*d* spacing (Å)	*T*_d_ (°C)
DAS-PI1	15.15, 25.04	5.84, 3.55	500
DAS-PI2	16.5	5.36	520
DAS-PI3	16.94	5.22	525

a 2*θ* and *d* spacing value obtained from WAXD curves. *T*_d_, temperature at which 10% weight loss was recorded by TGA.

High molecular weights and good mechanical properties of potential gas separation polymers are often indicative of their ability to be converted to a thin film configuration or hollow fibers; a requisite for gas separation module fabrication. Nano-indentation analysis reveals that the synthesized DAS-PI membranes have excellent mechanical properties. The average elastic modulus and hardness of the DAS-PIs are summarized in Table 3, and compared with several polymeric microporous materials in Table S3.† The DAS-PI membranes have hardness value ranging from 240 to 270 MPa and elastic modulus of 3 to 4.5 GPa. Interestingly, these membranes showed significantly comparable tensile strength as in KAUST-PI1, PIM-PI1 and conventional polyimides such as Matrimid®^24^ (Table S3†). Such a high tensile strength may generate from more entangled state and flexible backbone of polymer of DAS-PIs. The thermal properties of the polyimides were investigated by TGA and differential scanning calorimetry (DSC). DAS-PI 2 and DAS-PI3 membranes showed superior thermal stability up to 525 °C (*T*_d_, 10% mass loss), and DAS-PI1 membrane showed thermal stability up to 500 °C (*T*_d_, 10% mass loss). There was no observation of glass transition temperature (*T*_g_) up to 400 °C (Table 2, Fig. S3†). It suggests that DAS-PI3 and DAS-PI2 are mechanically and thermally more stable than DAS-PI1 because of their flexible backbone.

**Table 3:** Mechanical properties of DAS-PI polyimides^a^

Polymer	Hardness (MPa)	Elastic modulus (GPa)
DAS-PI1	243.7	3.68
DAS-PI2	237.6	4.59
DAS-PI3	271.2	4.27

a Average elastic modulus and hardness were calculated by Oliver and Pharr's method.

DAS-PIs membranes were successfully prepared for gas permeation tests. The transport properties of the DAS-PIs derived from DAS-diamine were compared with the separation data of previously reported PIs with similar dianhydride monomers to identify the effect of synthesis method (Table S4†). H_2_, N_2_, O_2_, CH_4_, and CO_2_ pure-gas permeation measurements were carried out at 25 °C and 4 bar (Table 4). The order of gas permeability is DAS-PI1 > DAS-PI2 > DAS-PI3. The high gas permeability of DAS-PI1 is due to increasing in fractional free volume (FFV) that can be ascribed to the rigidity and contorted structure of the polymer backbone itself. In addition, the presence of stiff hexafluoroisopropyl groups of 6FDA reduces intra-chain packing. This was also corroborated with the result from BET surface area. It is noteworthy that gas permeabilities data of DAS-PI1 are in a comparable range with the reported polyimides having 6FDA in their backbone such as PIM-PI3,^21^ PIM-6FDA-OH,^39^ 6FDA-SBF^33^ and 6FDA-DATRI^32^ (Table S4†). The gas permeabilities decrease in DAS-PI2 and DAS-PI3 membranes consistent with the FFV results as discussed above. The influence of structural variation in dianydride of DAS-PIs membrane on the selectivity of gas pairs is readily visualized as shown in Table 4. Despite the smaller surface areas and consequently lower permeabilities, DAS-PI2 and DAS-PI3 membranes have higher gas pair selectivity than DAS-PI1, indicating the typical Robeson-type trade-off between selectivity and permeability (Table 4). For example, the measured CO_2_/CH_4_ selectivity of DAS-PI3 membrane is 1.6 times higher than that of DAS-PI1 (38 *vs.* 23). Similarly, the measured H_2_/CH_4_ selectivity of DAS-PI3 membrane is 2.7 times higher that of DAS-PI1 (61 *vs.* 22). The gas separation properties of DAS-PIs made by our facile synthesis method here are in the same range as other reported PIM-PIs, such as PIM-6FDA-OH,^39^ 6FDA-SBF,^33^ and 6FDA-DATRI,^32^ (Robeson's upper bounds, ESI†).

**Table 4:** Gas permeation measurement of DAS-PI polyimides^a^

Permeability (barrer)						Ideal selectivity					
Polymer	H_2_	N_2_	O_2_	CH_4_	CO_2_	CO_2_/N_2_	CO_2_/CH_4_	H_2_/N_2_	H_2_/CH_4_	H_2_/CO_2_	O_2_/N_2_
DAS-PI1	313	16.5	61.4	14.4	333	20.2	23.1	19	22	0.94	3.7
DAS-PI2	191	5.5	29.4	5.7	158	28.7	27.6	34.7	33.5	1.2	5.3
DAS-PI3	123	2.5	15.2	2	76.3	30.5	38	49.2	61.5	1.6	6

a 1 Barrer = 10^−10^ cm^3^ (STP) cm cm^−2^ s^−1^ (cm Hg)^−1^ or 7.5 × 10^−18^ m^3^ (STP) m m^−2^ s^−1^ Pa^−1^. All gases measured at 4 bar and 25 °C.

In summary, we provide a convenient and versatile method for large scale synthesis of DAS diamine in two steps from commercially available TTSBI and 3,4-difluoronitrobenzene. Various microporous polyimides were synthesized (DAS-PIs) based on DAS diamine and commercial dianhydride. The gas separation performance, mechanical and thermal stabilities of the obtained polyimides are in the observed range for other PIM-PIs. The notable characteristic of this study is the simplicity of the synthesis pathway by providing an alternative route to achieve the same material.

Our developed method for synthesis of contorted DAS-diamine, requiring few synthetic steps and less purification procedures, providing a potential pathway for efficient synthesis of other diamine monomers and microporous polyimides such as KAUST-PIs and PIM-SBI-TB for gas separation applications.

## Conflicts of interest

There are no conflicts to declare.

## Supplementary Material

RA-008-C7RA12719G-s001
