# Supplementary material for: A facile synthesis of contorted spirobisindane-diamine and its microporous polyimides for gas separation
Source: RSC Adv. 2018 Feb 7;8(12):6326–30. doi: 10.1039/c7ra12719g (PMC9078231; doi:10.1039/c7ra12719g)
Supplement: RA-008-C7RA12719G-s001 [file RA-008-C7RA12719G-s001.pdf]

Electronic Supplementary Material (ESI) for RSC Advances.
This journal is © The Royal Society of Chemistry 2018

A Facile Synthesis of Contorted Spirobisindane-diamine and its Microporous Polyimides For Gas Separation

Binod Babu Shrestha,[a] Kazuki Wakimoto,[a] Zhenggong Wang,[a] Ali Pournaghshband Isfahani,[a] Tomoya Suma,[a] Easan Sivaniah,*[a] and Behnam Ghalei *[a]

Institute for Integrated Cell-Material Sciences (iCeMS)
Kyoto university
606-8501 Kyoto, Japan.
E-mail: esivanah@icems.kyoto-u.ac.jp; bghalei@icems.kyoto-u.ac.jp

Experimental Procedures

5,5′,6,6′-tetrahydroxy-3,3,3′,3′-tetramethyl-1,1′-spirobisindane was obtained from Wako. 4,4′-(Hexafluoroisopropylidene) diphthalic anhydride (6FDA), 3,3,′4,4′-Biphenyltetracarboxylic dianhydride (BPDA), 3,3,′4,4′-benzophenone tetracarboxylic dianhydride (BTDA) were obtained from TCI. 3,4 difluronitrobenzene and *m*-cresol were obtained from Aldrich. Isoquinoline was obtained from Nacalai and They were used as received. The chemical structures of monomer and polymer were characterized by ^{1}H NMR, ^{13}C NMR, FT-IR and MALDI MASS. ^{1}H and ^{13}C NMR spectra were measured on Bruker AVANCE III 500 NMR spectrometer spectrometers at 23 °C respectively. $CDCl_3$ and DMSO-d_6 were used as a solvent and the residual solvent peaks were used as an internal standard (^{1}H NMR: $CDCl_3$ 7.26 ppm, DMSO-d_6 2.5 ppm; ^{13}C NMR: $CDCl_3$ 77.0 ppm, DMSO-d_6 39.52 ppm). The IR spectrum was collected in a. Fourier Transform Infrared spectrometer (FTIR, Shimadzu, IRTracer- 100), equipped with an attenuated total reflectance (ATR) cell in the range of 4000–500 cm^{-1}. The crystalline structure of membrane was characterized using wide-angle X-ray diffraction (WAXD, Rigaku RINT, Japan) with rotating-anode Cu Kα X-ray generator operated at 200 mA and 40 kV. Gel permeation chromatography (GPC, Shimadzu, 800 series) was used to analyze the molecular weight and polydispersity index of the synthesized polymer relative to polystyrene standards. MALDI-TOF mass spectroscopy was performed with the Microflex system (Microflex-ks II, Bruker). Differential scanning calorimetry (DSC) (DSC-60Plus, SHIMADZU) measurements were carried out at a heating rate of 10 °C/min under a nitrogen atmosphere. Thermo-gravimetric analysis (TGA, Rigaku Thermo plus EVO2, Japan) was utilized under a nitrogen atmosphere at a 10 °C/min ramp rate to evaluate membrane. Mechanical property tests were performed on the surface of polymer membranes using a nanoindentation tester (ENT 2100, Elionix) equipped with a Berkovich three-sided pyramid diamond tip (radius of 100 nm) at the load of 50 mN. 20 points in a rectangular configuration were tested on each sample; the average elastic modulus and hardness were calculated based by Oliver and Pharr's method using the measured values of three different membrane samples. Pure gas permeability of the membranes were tested at room temperature.

Geometric modeling

The ground state geometric structures of 3,3,3',3' tetramethyl-8,8-diamino-2,2',3,3'-tetrahydro-1,1'-spirobi [cyclopentalb|dibenzo[b,e][1,4]dioxine (SBI-diamine), **3** and 8,8′,9,9′-Tetrahydro-9,9,9′,9′-tetramethyl-7,7′-spirobis [7H-cyclopent[g]isobenzofuro[5,6-b][1,4]benzodioxin]-1,1',3,3'-tetrone (SBI-dianhydride), **An-1**[1] were fully optimized by the density hybrid function (B3LYP) method with 6-31G(d) as basis set, and calculations were carried out using the Gaussian 09 software package.

Membrane fabrication

Free standing membrane of synthesized polymer were prepared by solution casting and thermal phase inversion method. The polymer DAS-PI1 were dissolved in chloroform (5 wt %/v) and then purified using small 5.0 µm PTFE filter cartridges. The solution was carefully transferred into a glass petri-dish and covered glass petri-dish; thereafter, the solvent was evaporated slowly over two days at room temperature. The dry isotropic polymer films (50 to 60 µm thick) were soaked in methanol for 24 h, air-dried and then heated at 120 °C for 24 h under high vacuum. In the case of DAS-PI2 and DAS-PI3, They were dissolved in NMP (5 wt %/v) and then purified using small 5.0 µm PTFE filter cartridges. The solution was carefully transferred into a glass petri-dish and covered by grated aluminium foils; thereafter, the solvent was evaporated slowly over three days at 70 °C temperature. The dry isotropic polymer films (50 to 60 µm thick) were soaked in methanol for 24 h for solvent exchange with NMP and finally optimized drying condition by keeping membrane at 120 °C under vacuum for 24 h. There was no change in weight of membrane by keeping at 120 °C under vacuum after 24 h which was further confirmed by thermogravimetric analysis (TGA).

Gas separation measurements

Pure gas permeabilities of the membranes were determined using the constant pressure-variable volume method at room temperature (25 ºC). The membrane was held in a Millipore commercial filter holder with steel meshed supports, and rubber O-rings were used for proper sealing. The gas permeate pressure was recorded by pressure transmitters (Keller PAA 33X) connected to a data acquisition system. The slope of pressure increase (*dp*/*dt*) in the permeate chamber became constant at the pseudo-steady state. The gas permeability (*P*) is calculated based on the following equation:

$$P = \frac{Vl}{A} \frac{T_0}{p_f p_0 T} (\frac{dp}{dt}) \qquad \text{------------(1)}$$

where P is the permeability of the gas through the membrane, in Barrer (1 Barrer = 10^{-10} cm^3(STP)cm·cm^{-2}·s^{-1}·$cmHg^{-1}$), V is the permeate volume (cm^3), l is the thickness of membrane (cm), A is the effective area of the membrane (cm^2), p_f is the feed pressure (cm-Hg), p_0 is the pressure at standard state (76 cm-Hg), T is the absolute operating temperature (K), T_0 is the temperature at standard state (273.15 K), (dp/dt) is the slope of pressure increase in the permeate volume at pseudo-steady state (cm-Hg/s).

The ideal selectivity (α) of gas pairs, A and B, is defined as:

$$\alpha_{A/B} = \frac{P_A}{P_B} = \left[\frac{D_A}{D_B}\right]\left[\frac{S_A}{S_B}\right] \text{-----------------(2)}$$

where D_A/D_B is the diffusivity selectivity and S_A/S_B is the solubility selectivity.

The feed side pressure of the gas is 4 bar.

Synthesis of Dinitro-SBI (**2**): 3,4-Difluoronitrobenzene (0.126 mol, 20.04 g) and 5,5′,6,6′-tetrahydroxy-3,3,3′,3′-tetramethyl-1,1′-spirobisindane (0.0599 mol, 20.4 g) were stirred under N_2. Dry dimethyl-formamide (250 mL) and K_2CO_3 (33 g) were added slowly, and then the mixture was stirred for 2 h at room temperature then gradually increased temperature up to 110 °C and kept at 110 °C for 12 h. The product was isolated, washed with distilled water, dried, and recrystallized from methanol (yield, 95%). ^{1}H NMR (500 MHz, $CDCl_3$): δ 1.32 (s, 6H, Me), 1.37 (s, 6H, Me), 2.18 (d, 2H, J = 12.8 Hz, CH_2), 2.33 (d, 2H, J = 12.8 Hz, CH_2), 6.35 (s, 2H, Ar-H), 6.69 (s, 2H, Ar-H), 6.82 (d, 1H, J = 7.8 Hz, Ar-H), 6.91 (d, 1H, J = 7.8 Hz, Ar-H), 7.62 (d, 1H, J = 2.5 Hz, Ar-H), 7.71 (d, 1H, J = 2.5 Hz, Ar-H), 7.77-7.82 (m, 2H, Ar-H). ^{13}C-NMR (125 MHz, $CDCl_3$) δ 30.06, 31.39, 31.4, 43.38, 43.42, 57.12, 59.06, 109.99, 110.02, 110.04, 110.06, 111.87, 111.92, 112.13, 112.21, 116.19, 116.26, 119.95, 140.38, 140.44, 140.5, 140.54, 142.13, 143.56,145.95, 145.99, 146.35, 146.38, 147.55, 147.6, 148.57, 148.59, 148.99. MALDI Mass [M^+]: m/z 577. 874, ATR-IR (ν, cm^{-1}): 2947 (str C−H), 1489 (asym str, N=O), 1315 (sym str, N=O).

Synthesis of SBI-diamine, **3** (DAS-amine): To a dispersion of **2** (12 g, 0.02 mol) in ethanol (500 mL) was added 10% palladium on charcoal (1.0 g); hydrazine monohydrate (10 mL) was added to the stirred mixture dropwise at 85 °C for 15 min. After addition was completed, the mixture was stirred at 85 °C for another 8 h. The solution was then filtered to remove Pd/C. The crude product was recrystallized from 95% ethanol and water to give off white powder: yield (84%). ^{1}H NMR (500 MHz, DMSO-d_6): δ 1.25 (s, 6H, Me), 1.32 (s, 6H, Me), 2.09 (d, 2H, J = 12.9 Hz, CH_2), 2.24 (d, 2H, J = 12.9 Hz, CH_2), 4.95 (br, 4H NH_2), 6.11-6.14 (m, 3H, Ar-H), 6.17 (s, 1H, Ar-H), 6.20 (s, 1H, Ar-H), 6.25 (s, 1H, Ar-H), 6.55 (d, 1H, J = 7.5 Hz, Ar-H), 6.64 (d, 1H, J = 7.5 Hz, Ar-H), 6.77 (s, 1H, Ar-H), 6.82 (s, 1H, Ar-H). ^{13}C-NMR (125 MHz, DMSO-d_6) δ 30.29, 31.57, 43.29, 43.30, 56.99, 57.01, 57.04, 59.14, 101.9, 101.93, 109.17, 109.2, 109.96, 110.17, 111.25, 111.44, 116.71, 132.18, 132.2, 141.23, 141.35, 141.95, 142.01, 142.08, 144.86, 144.9, 145.37, 145.42, 145.93, 145.95, 147.21, 147.22, 147.71. MALDI MASS [M^+]: 517. 742. ATR-IR (ν, cm^{-1}): 3460 (str NH_2), 2947 (str C−H), 1641 (NH_2), 1504 (str NH_2), 1330 (str C-N).

Synthesis of DAS-PI1 (**4a**): DAS-diamine(**3**) (1.03 g, 2 mmol) and 6FDA (0.88 g, 2 mmol) were added to *m*-cresol (10 mL). The solution was stirred at room temperature for 3 h before isoquinoline (0.2 mL) was added. The solution was then heated gradually to 180 °C and kept for 5 h. During this period, water was removed from the reaction mixture by azeotropic distillation and adding 1 mL toluene. The resulting polymer was then precipitated in methanol (200 mL), and the remaining *m*-cresol in the polymer was removed by Soxhlet extraction. The polymer was dried and then dissolved in chloroform and reprecipitated in methanol two times. Finally, the polymer was dried in a vacuum oven at 150 °C for 24 h and a light yellow filament polymer was obtained. (yield: 94%). ^{1}H NMR (500 MHz, $CDCl_3$): δ 1.31 (s, br, 6H, Me), 1.35 (s, br, 6H, Me), 2.18 (d, br, 2H, J = 12.7 Hz, CH_2), 2.31 (d, br, 2H, J = 12.7 Hz, CH_2), 6.35 (s, br, 2H, Ar-H), 6.66 (s, br, 2H, Ar-H), 6.86-6.94 (m, br, 6H, Ar-H), 7.86-8.01 (m, br, 6H, Ar-H). ATR-IR (polymer film, ν, cm^{-1}): 2947 (str C−H), 1759 (asym str, C=O, imide), 1728 (sym str, C=O, imide), 1327 (C-N str), 721 (amide ring deformation). Molecular weight: Mw =95×10^3 g/mol; PDI=1.4, T_d = 500°C (10% mass loss), BET surface area: S_{BET} = 345 m^2/g.

Synthesis of DAS-PI2 (**4b**): The synthetic procedure was the same as that described for DAS-PI1; the polymer was obtained as a yellow powder with a yield of 96%. ATR-IR (polymer film, ν, cm^{-1}): 2941 (str C−H), 1776 (asym str, C=O, imide),), 1724 (sym str, C=O, imide),1319 (C-N str), 736 (amide ring deformation).T_d = 520 °C (10% mass loss), BET surface area: S_{BET} = 43 m^2/g.

Synthesis of DAS-PI3 (**4c**): The synthetic procedure was the same as that described for DAS-PI1; the polymer was obtained as a dark yellow powder with a yield of 92%. ATR-IR (polymer film, ν, cm^{-1}): 2953 (str C−H), 1778 (asym str, C=O, imide), 1722 (sym str, C=O, imide), 1321 (C-N str), 740 (amide ring deformation). T_d = 525 °C (10% mass loss), BET surface area: S_{BET} = 17 m^2/g.

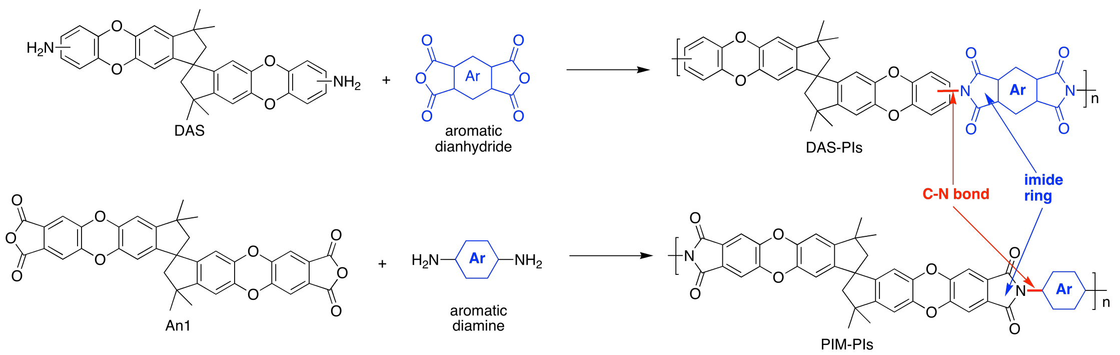

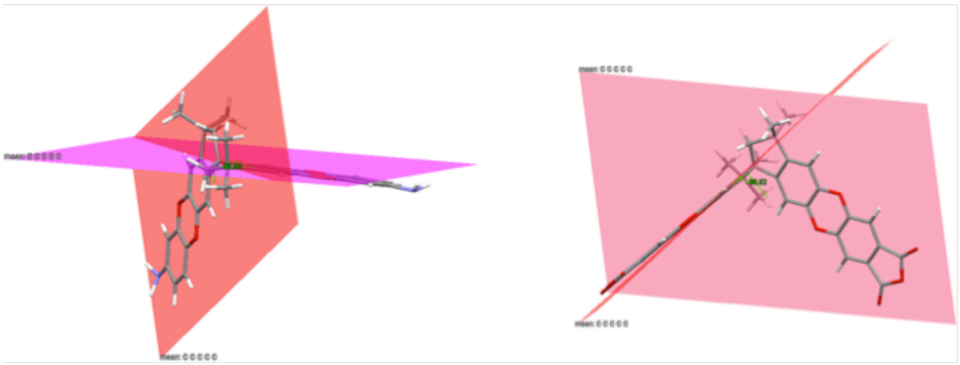

DAS (contortion angle = 89.5 °

An1 (contortion angle = 89.5 °

Figure S1: Structural difference between DAS-PIs and PIM-PIs and their monomers

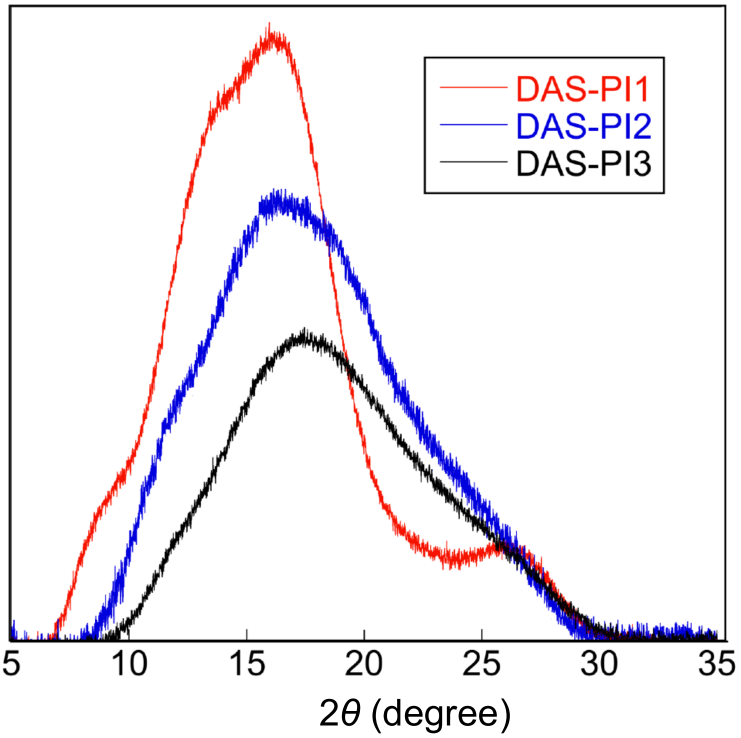

Figure S2: WAXD pattern of DAS-PIs membrane

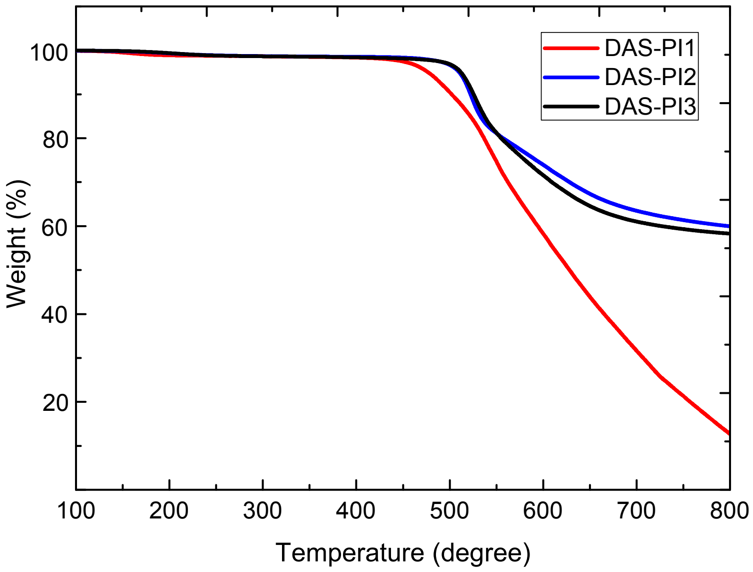

Figure S3: TGA pattern of DAS-PIs membrane

Table S1: Molar masses, surface areas and TGA of DAS-PIs[a]

Polymer	M_n[g/mol]	M_w[g/mol]	M_w/M_n	T_d	S_{BET}
DAS-PI1	66 000	95 000	1.4	500	345
DAS-PI2	-	-	-	520	43
DAS-PI3	-	-	-	525	17

[a]Number-average molar mass, M_n, weight-average molar mass, M_w, and polydispersity, M_w/M_n, determined by GPC (refractive index detectors). BET surface area, S_{BET}, determined from N_2 adsorption at 77 K. Thermo-gravimetric analysis (TGA, T_d = 10% weight loss) was utilized under a nitrogen atmosphere at a 10 °C/min ramp rate to evaluate membrane.

Table S2: Solubility of DAS-PIs in Various Solvents[a]

Polymer	$CHCl_3$	THF	m-cresol	acetone	DCM	DMA	NMP
DAS-PI1	++	++	++	-	++	+-	+-
DAS-PI2	-	-	++	-	-	-	++
DAS-PI3	-	-	++	-	-	-	++

[a]Qualitative solubility was determined with 5 mg of polymer in 0.5 mL solvent at room temperature: ++, completely dissolved; - insoluble; +-, partially soluble; THF: tetrahydrofuran; DCM: dichloromethane; NMP: N-methyl-2-pyrrolidone; DMA: N, N-dimethylacetamide.

Table S3: Mechanical properties of DAS-PI polyimides

Polymer	Hardness (MPa)	Elastic Modulus (GPa)
DAS-PI1	243.7	3.68
DAS-PI2	237.6	4.59
DAS-PI3	271.2	4.27
PIM-PI1	-	1.62[2]
KAUST-PI1	-	2.46[2]
Matrimid	-	2.89[2]

Average elastic modulus and hardness were calculated based by Oliver and Pharr's method. [2]reference

Table S4: Gas permeation measurement of DAS-PI and other PIM-polyimides

	Permeability (barrer)					Ideal selectivity					
Polymer	H_2	N_2	O_2	CH_4	CO_2	CO_2/N_2	CO_2/CH_4	H_2/N_2	H_2/CH_4	H_2/CO_2	O_2/N_2
DAS-PI1	313	16.5	61.4	14.4	333	20.2	23.1	19	22	0.94	3.7
DAS-PI2	191	5.5	29.4	5.7	158	28.7	27.6	34.7	33.5	1.2	5.3
DAS-PI3	123	2.5	15.2	2	76.3	30.5	38	49.2	61.5	1.6	6.0
PIM-PI3[1]	360	23	85	27	520	23	19	16	13	1.4	3.7
PIM-6FDA-OH[3]	259	11	45	9	263	24	29	24	29	1.0	4.2
6FDA-DATRI[4]	257	8.1	39	6.2	189	23	31	32	42	1.4	4.8
6FDA-SBF[5]	234	7.8	35	6.4	182	23	27	30	37	1.3	4.5
6FDA-BSBF[5]	531	27	107	25	580	22	23	20	21	1.1	4.0

[1,3,4,5] reference

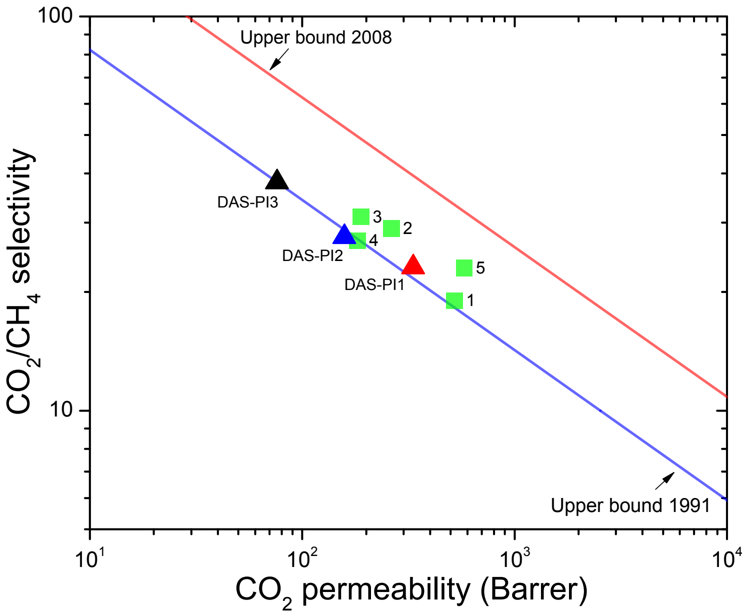

Figure S4: Relationship between gas permeability (P) and gas pair selectivity (α) with Robeson upper bound for CO_2/CH_4 red : DAS-PI1; blue: DAS-PI2; black: DAS-PI3 and green: **1** to **5**. (**1**: PIM-PI3[1]; **2**: PIM-6FDA-OH[3]; **3**: 6FDA-DATRI[4]; **4**: 6FDA-SBF[5] and **5**: 6FDA-BSBF[5].

Reference

1. B. S. Ghanem, N. B. McKeown, P. M. Budd, N. M. Al-Harbi, D. Fritsch, K. Heinrich, L. Starannikova, A. Tokarev and Y. Yampolskii, *Macromolecules,* 2009, **42**, 7881-7888.
2. R. Swaidan, M. Al-Saeedi, B. Ghanem, E. Litwiller and I. Pinnau, *Macromolecules,* 2014, **47**, 5104-5114.
3. X. Ma, R. Swaidan, Y. Belmabkhout, Y. Zhu, E. Litwiller, M. Jouiad, I. Pinnau and Y. Han, *Macromolecules,* 2012, **45**, 3841-3849.
4. Y. J. Cho and H.B. Park, *Macromol. Rapid Commun.,* 2011, **32**, 579-586.
5. X. Ma, O. Salinas, E. Litwiller and I. Pinnau, *Macromolecules,* 2013, **46,** 9618-9624.
